# Optimization of high throughput spectral flow cytometry for immune cell profiling in mouse liver^☆^

**DOI:** 10.1016/j.livres.2023.08.001

**Published:** 2023-08-07

**Authors:** Grayson W. Way, Hongkun Lu, Xuan Wang, Derrick Zhao, Carmen Camarena, Devanand Sarkar, Rebecca K. Martin, Huiping Zhou

**Affiliations:** a C. Kenneth and Dianne Wright Center for Clinical and Translational Research, Virginia Commonwealth University, Richmond, VA, USA; b Department of Microbiology and Immunology, Virginia Commonwealth University School of Medicine, Richmond, VA, USA; c Department of Human and Molecular Genetics, Massey Cancer Center, VCU Institute of Molecular Medicine (VIMM), Virginia Commonwealth University, Richmond, VA, USA; d Massey Cancer Center, Virginia Commonwealth University, Richmond, VA, USA; e VCU Institute of Molecular Medicine, Virginia Commonwealth University, Richmond, VA, USA; f Central Virginia Veterans Healthcare System, Richmond, VA, USA

## Abstract

The liver plays an important role in both metabolism and immunity. Disruption of the hepatic immune microenvironment is closely associated with various liver diseases. To gain a better understanding of how different types of immune cells contribute to the progression of liver diseases, it is crucial to thoroughly characterize hepatic immune cells. Although direct digestion of liver tissue is a relatively simple method for isolating immune cells, it often induces excessive hepatocyte death, which causes a release of intracellular components that leads to the activation of stress responses and injury in the surrounding cells. This injury can lead to excessive death in the hepatic immune cells, making isolation and accurate characterization of the immune profile challenging, especially in diseased livers. The method described here addresses these challenges by utilizing Phosphate buffered saline (PBS) and digestion buffer perfusions to eliminate contaminating blood cells, ensure a pure hepatic immune population, and minimize hepatic immune cell death. Further *ex vivo* digestion of the liver enables the isolation of the immune cells from the hepatic tissues and the generation of a single-cell suspension that can be stained for spectral flow cytometry. To enhance intracellular cytokine detection and maintain signaling under different physiological and pathological conditions, this protocol uses an *in vivo* administration of Brefeldin A, a less toxic inhibitor of cytokine secretion. This *in vivo* administration of Brefeldin A allows for a more accurate representation of the immune cell function and cytokine expression compared to the traditionally used *ex vivo* Brefeldin A administration. A comprehensive spectral flow cytometry panel, comprising extracellular and intracellular staining, is used for deep immunophenotyping and immune cell effector function profiling. While this protocol is specifically designed for liver digestion of *Mdr2* knockout mice (a model for primary sclerosing cholangitis) and flow cytometry staining, it can also be applied to other liver diseases and sensitive tissues.

## Graphical abstract

**Figure:**
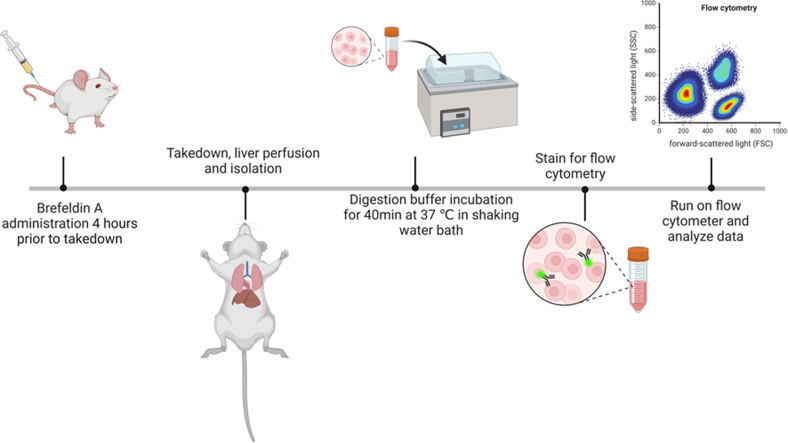

## 1 Introduction

Accurate and comprehensive identification of pure hepatic immune cells is crucial for understanding their role in the progression of various liver diseases. The methods described here provide a high throughput immunophenotyping panel and methodology developed and validated by our lab for this purpose. Previously published methods in hepatic immunophenotyping have been limited in their ability to accurately characterize the hepatic immune compartment, identify immune cell effector functions, and incorporate cytokine staining.^1^ This study addresses these limitations and takes advantage of the advancements in spectral flow cytometry. Traditional flow cytometry approaches had limitations in the number of markers that could be simultaneously analyzed. However, with spectral flow cytometry, the analysis of a larger number of fluorophores and markers is feasible, allowing for more comprehensive identification of immune cells in a single run. The methods described here take advantage of the increased capabilities of spectral flow cytometry to provide an in-depth immune panel and methodology that can be applied to identify pathological immune changes associated with several liver diseases. While extracellular surface staining can help identify different cell types in these disease states, intracellular cytokine staining is crucial for determining immune cell effector functions and their interaction with surrounding cells. However, cytokine staining for flow cytometry can be challenging due to the continuous export of cytokines from active immune cells, which can weaken the signal during analysis. To overcome this, our method utilizes *in vivo* treatment with Brefeldin A, which increases cytokine staining while preserving the native disease state signaling. This approach allows us to elucidate the true effector function of hepatic immune cells without artificial activation by phorbol 12-myristate 13-acetate (PMA)/ionomycin.^2^

## 2 Preparation

Before you begin, the investigator needs to be familiar with the techniques utilized in this protocol. Specifically, the investigator should be familiar with mouse handling, tail vein injection, liver perfusion, cell isolation, and large panel spectral flow cytometry techniques involving both surface and intracellular staining. Table 1 provides all key resources, and Table 2A, Table 2B, Table 2C, Table 2DA–D list all materials and equipment required for the procedure.

**Table 1:** Key resources table.

**Reagent or resource**	**Source**	**Cat#**	**Lot**	
**Antibodies**				
Spark Blue™ 550 anti-mouse CD45 (clone 30-F11)	BioLegend	103166	B368696	
BD Horizon™BB700 rat anti-human/mouse CD11b (clone M1/70)	BD Biosciences	566416	1350969	
PE-Cyanine5 rat anti-mouse F4/80 (clone BM8)	Thermo Fisher Scientific	15-4801-82	2349883	
APC rat anti-mouse Ly-6C (clone HK1.4)	BioLegend	128016	B353568	
PerCP-eFluor™ 710 rat anti-mouse Tim4 (clone 54 (RMT4-54))	Thermo Fisher Scientific	46-5866-82	2294211	
PE rat anti-mouse CLEC-2 (clone 17D9/CLEC-2)	BioLegend	146104	B337666	
eFluor™ 450 rat anti-mouse MHC Class II (I-A/I-E) (clone M5/114.15.2)	Thermo Fisher Scientific	48-5321-82	2309275/2461740	
Brilliant Violet (BV)™ 510 Armenian hamster anti-mouse CD11c (clone N418)	BioLegend	117337	B338021	
BV 785 rat anti-mouse CD335 (NKp46) (clone 29A1.4)	BioLegend	137637	B347194	
AlexaFluor (AF)™ 700 rat anti-mouse Ly-6G (clone 1A8-Ly6g)	Thermo Fisher Scientific	56-9668-82	2538347	
Super Bright™ 702 mouse anti-mouse NK1.1 (cone PK136)	Thermo Fisher Scientific	67-5941-82	2472731	
APC-eFluor™780 rat anti-mouse CD90.2 (clone 53–2.1)	Thermo Fisher Scientific	47-0902-82	2371181	
Brilliant Ultra Violet (BUV)™ 737 Armenian hamster anti-mouse TCR beta (TCRβ, clone H57-597)	Thermo Fisher Scientific	367-5961-82	2546931	
PerCP rat anti-mouse CD4 (clone GK1.5)	BioLegend	100432	B352993	
BUV805 rat anti-mouse CD8a (clone 53–6.7)	Thermo Fisher Scientific	368-0081-82	2400301/2682414	
PE/Cyanine7 rat anti-mouse CD304 (clone 3E12)	BioLegend	145212	B309196/B370165	
NovaFluor™ Blue 585 rat anti-mouse CD62L (clone MEL-14)	Thermo Fisher Scientific	M006T02B04	2486370/2561879	
BV570 rat anti-mouse CD44 (clone IM7)	BioLegend	103037	B361473/B376458	
BD OptiBuild™ BV750 Armenian hamster anti-mouse γδ T-Cell Receptor (TCRγδ) (clone GL3)	BD Biosciences	746962	2315084	
BV605 rat anti-mouse interleukin(IL)-10 (clone JES5-16E3)	BioLegend	505031	B348011/B384759	
Spark NIR™ 685 rat anti-mouse IFN-γ (clone XMG1.2)	BioLegend	505861	B333425/B379291	
BV421 rat anti-mouse CD206 (clone C068C2)	BioLegend	141717	B361008	
PE-eFluor™ 610 rat anti-mouse TNF-alpha (TNF-α, clone MP6-XT22)	Thermo Fisher Scientific	61-7321-82	2459621	

**Chemicals, buffers, peptides, enzymes, and recombinant proteins**				
Zombie UV™ Fixable Viability Kit	BioLegend	423108	B342952	
Mouse BD Fc Block	BD Biosciences	553142	2129958	
Collagenase Type 4	Worthington	LS004189	42C22290	
Deoxyribonuclease I	Bioland Scientific	EPD01-02	31323425Q	
Isoflurane				
70% EtOH				
Intracellular Staining Permeabilization Wash Buffer	BioLegend	421002	B350872	
Brilliant Stain Buffer	BD Biosciences	566349	1281962	
RPMI 1640	Gibco	11875119	2508211	
ACK Lysing Buffer	Gibco	A1049201	2393369	
Fixation Buffer	BioLegend	420801	B356163	

**Experimental models: organisms/strains**				
Mouse: *Mdr2*^*−/−*^: FVB	The Jackson Laboratory	JAX# 002539		

**Software and algorithms**				
FlowJo v10.8.1	FlowJo	N/A		

**Spectral flow cytometer used**				
5 laser Cytek® Aurora	Cytek®	N/A		

Others include 6-well culture plate, 70 μm tube top cell strainer, 50 mL conical tubes, Syringe pump, Oxygen tank, Erlenmeyer flasks, Syringe pump tubing, 20-gauge cannula, Gauze, Catheter, Shaking water bath, Centrifuge, Vortexer, Micropipettes, Transfer pipettes, Scalpel and Forceps.

**Table 2A:** Materials and equipment: extracellular antibody master mix.

**Antibody or diluent**	**Volume**
SparkBlue 550 anti-mouse CD45	1 μL per 100 μL volume
BB700 rat anti-human/mouse CD11b	1 μL per 100 μL volume
PE-Cyanine5 anti-mouse F4/80	1 μL per 100 μL volume
APC anti-mouse Ly-6C	1 μL per 100 μL volume
PerCP-eFluor710 anti-mouse Tim4	1 μL per 100 μL volume
PE anti-mouse CLEC-2	1 μL per 100 μL volume
eFluor450 anti-mouse MHC Class II (I-A/I-E)	1 μL per 100 μL volume
BV510 anti-mouse CD11c	1 μL per 100 μL volume
AF700 anti-mouse Ly-6G	1 μL per 100 μL volume
Super Bright 702 anti-mouse NK1.1	1 μL per 100 μL volume
BV785 anti-mouse CD335	1 μL per 100 μL volume
APC-eFluor780 anti-mouse CD90.2	1 μL per 100 μL volume
BUV737 anti-mouse TCRβ	1 μL per 100 μL volume
PerCP anti-mouse CD4	1 μL per 100 μL volume
BUV805 anti-mouse CD8a	1 μL per 100 μL volume
PE/Cyanine7 anti-mouse CD304	1 μL per 100 μL volume
NovaFluor Blue 585 anti-mouse CD62L	1 μL per 100 μL volume
BV570 anti-mouse CD44	1 μL per 100 μL volume
BV750 anti-mouse TCRγδ	1 μL per 100 μL volume
CellBlox blocking buffer	5 μL per 100 μL of cell sample
Brilliant stain buffer	50 μL per 100 μL volume
Flow cytometry (FACS) buffer	Add until the total volume is 500 μL (per sample)

CellBlox blocking buffer is shipped with each NovaFluor conjugate or kit. If the cell count is too high, volume can be increased by 100 μL increments until desired cell concentration is reached. The ideal cell number per 100 μL is 1 million cells. CD90.2 will not stain lymphocytes for FVB background mice. It is included in this panel as this panel is also used successfully in our *Mdr2*^*−/−*^ mice with C57BL/6 backgrounds.

**Table 2B:** Materials and equipment: intracellular antibody master mix.

**Antibody or diluent**	**Volume**
BV605 anti-mouse IL-10	1 μL per 100 μL volume
SparkNIR 685 anti-mouse IFN-γ	1 μL per 100 μL volume
BV421 anti-mouse CD206	1 μL per 100 μL volume
PE-eFluor610 anti-mouse TNF-α	1 μL per 100 μL volume
Brilliant stain buffer	50 μL
Intracellular staining permeabilization wash buffer	Add until the total volume is 500 μL (per sample)

**Table 2C:** Materials and equipment: FACS buffer.

**Reagent**	**Final concentration**
Ethylenediamine tetraacetic acid (EDTA)	2 mmol/L
Bovine serum albumin (BSA)	2%
Phosphate buffered saline (PBS)	N/A
Sodium azide	0.05%–0.10%
Deoxyribonuclease I	25–50 μg/mL
Total	N/A
Store at 4 °C	

Alternatives: BSA can be replaced with fetal bovine serum. Deoxyribonuclease I is not required for FACS buffer but is recommended as it can mitigate excessive cell death seen during final analysis.

**Table 2D:** Materials and equipment: digestion buffer.

**Reagent**	**Final concentration**
Warmed RPMI 1640	
Collagenase type 4	1 mg/mL
Deoxyribonuclease I	≥40 μg/mL
Penicillin-streptomycin	1%
HEPES	1%
**Total**	**50 mL (per liver)**

Alternatives: The digestion buffer formula above assumes the use of high-quality deoxyribonuclease I. However, utilizing bulk deoxyribonuclease I has been used in this protocol (data not shown). In this alternative method, a large scoop of deoxyribonuclease I is added to the media until a color change is observed, indicating sufficient enzyme activity.

## 3 Step-by-step method details

### 3.1 Intravenous injection administration of Brefeldin A, 4 hours prior to the takedown

Prior to liver harvest, it is important to set up the surgical environment. Make sure that all necessary equipment, including syringes, reagents, buffers, etc., are ready in advance. In our experiments, we found that treating mice with Brefeldin A for 4 hours before hepatectomy yielded effective cytokine staining. However, it is important to note that different experimental procedures may have specific time requirements that should be considered accordingly.

**Step 1**. Four hours before mouse surgery, inject mice via tail veins with 100 μL of 2.5 mg/mL Brefeldin A resuspended in PBS.
  Tip: To enhance the ease and efficiency of tail vein injections, consider heating the mouse and tail vein under a heat lamp or with a heating pad. This can help promote vasodilation and facilitate the procedure. In addition, it is essential to ensure the proper sanitation of the injection site before treatment to minimize the risk of contamination.
  Note: The optimal dosage and timing of Brefeldin A administration have not been fully optimized in this protocol. The concentration and volume of Brefeldin A were modified from Kovacs *et al.*,^2^ to keep the same total amount of Brefeldin A while reducing the volume of tail vein injection volume. Kovacs *et al.*^2^ utilized a 6 hours timepoint for their peripheral blood mononuclear cell (PBMC) investigation; however, we observed strong interferon-gamma (IFN-γ) staining in our natural killer (NK) and natural killer T (NKT) cells at a 4 hours timepoint.^2^ The appropriate timepoint for Brefeldin A treatment may vary depending on specific cytokines of interest. It is essential to ensure that the injection volume and Brefeldin A treatment comply with your institution's Institutional Animal Care and Use Committee (IACUC) guidelines.
  Alternative: As elaborated in problem 2 of the troubleshooting section, alternative Golgi blockers may be used and may be more effective depending on the cytokines under investigation.

### 3.2 Anesthetization, followed by liver perfusion and organ isolation

Timing: 25–35 min per mouse.

**Step 2**. Prepare the digestion and FACS buffers. Prior to the surgery, ensure the digestion buffer and PBS that will be used for perfusion are kept warm in an incubator or bead bath/water bath held at 37 °C. This helps maintain the enzymes active during perfusion and digestion while also preventing temperature shock to the mice during the perfusion process.
**Step 3**. Set up the syringe pump.
  a. Syringe pumps should be set to a pump speed of 3 mL/min.
  b. A bottle containing 30 mL of PBS and another separate bottle containing 30 mL of digestion buffer should be made for each mouse and placed in the 37 °C warm water bath.
**Step 4**. Anesthetize the animal using isoflurane and oxygen inhalation. Once the mouse shows no response to the toe pinch, secure it onto the surgical board. Spray the abdominal area with 70% ethanol (EtOH) (and/or iodine) and surgically open the abdominal cavity to expose the liver and inferior vena cava.
**Step 5**. Cannulate the inferior vena cava approximately 5–6 mm below the entrance to the liver with an appropriate gauge needle (20G) and 1.25 inch catheter with the bevel side facing up. Carefully remove the inner needle and simultaneously insert the catheter approximately 3–4 mm into the vein. Adjust the positioning as needed to ensure blood flow is observed from the cannula. The catheter should move freely within the vein.
  c. Begin perfusing with PBS.
  d. After 2 to 3 seconds of perfusion, white spots on the liver should be appreciable. At this point, cut the portal vein and proceed to monitor the efficacy of liver perfusion by observing the gradual loss of color in the liver.
  e. Once the PBS has been depleted, stop the pump apparatus, and transfer the perfusion tubing to the bottle containing the digestion buffer and perfuse the liver until the bottle is empty.
  Note: If there are indications that the liver is not uniformly perfusing (such as certain areas maintaining a dark or red color during perfusion), gently pinching the portal vein with forceps can increase the pressure and fluid buildup in the liver. This technique may assist in perfusing those challenging areas.
**Step 6**. Once the perfusion is complete, remove the liver from the animal. Carefully remove and discard the gallbladder. Place the isolated liver in a 6-well plate with 5 mL RPMI 1640 on ice to wait until the rest of the mice are finished.
**Step 7**. Repeat steps 2 through 6 until livers have been isolated from each mouse.
  Note: A more in-depth liver perfusion methodology with surgical and equipment arrangement pictures can be found in Mendoza *et al.*^3^ or Charni-Natan *et al.*^4^ As described in Charni-Natan *et al.*,^4^ the outlined perfusion procedure allows for retrograde perfusion of the liver.
  Alternative: anesthetize animals. Once they are unresponsive to toe pinch, pin them to the board and expose the heart. Insert a butterfly needle into the left ventricle and connect it to the large 30 mL syringe filled with PBS, which is attached to a syringe pump. Set the pump to infuse at a rate of 6 mL/min for a total of 15 mL. Cut the right atrium. Transfer the butterfly connection to a second syringe pump containing the digestion buffer. Start the second syringe pump. Be careful not to disturb the positioning of the butterfly needle in the ventricle. Perfuse 30 mL digestion buffer at a rate of 2 mL/min. While manual perfusion with syringes is possible, it is not recommended. In addition, it is important to avoid perfusing too quickly.

### 3.3 Manual and enzymatic digestion, followed by sequential centrifugation to remove hepatocytes

Timing: 45–55 min.

Digest the livers mechanically and enzymatically to release the hepatic immune cells from the liver tissue and get them into solution.

**Step 8**. Once all livers have been isolated. Transfer each liver to an individual large weigh boat and vigorously chop them with a clean razor blade (ensure you are chopping and not smearing). Carefully mince the livers as fine as possible.
  Note: Chopping the liver is a very critical part of the protocol. Be sure not to smear the tissues.
**Step 9**. Scrape tissue together and use 5 mL of warmed digestion buffer to transfer the liver to a 50 mL conical tube (or Erlenmeyer flasks). Fill each container with 10 to 20 mL of warmed digestion buffer. Be sure it is consistent between your samples.
**Step 10**. Transfer the liver samples to a 37 °C shaking water bath. Set the shaking water bath to 100 rpm and allow the digestion to continue for 40 min.
  Note: Depending on the disease model and the level of fibrosis at the time of liver isolation, increasing the digestion time may be required to help release more immune cells from the hepatic tissues. Be aware that over-digestion could also reduce or remove important immune antigens. CD4 is especially sensitive to enzymatic digestion.

### 3.4 Preparation for flow cytometry staining

Timing: 20–25 min.

Create a single-cell suspension in preparation for flow cytometry staining.

**Step 11**. Remove livers from the incubator and manually grind through a 70 μm tube top cell strainer into a 50 mL conical tube. Remove the cell strainer and fill the tube with 45 mL ice-cold PBS (Keep on ice as hepatocytes are prone to apoptosis, and this will kill your other cells as they die and release deoxyribonucleic acid (DNA).
**Step 12**. Centrifuge the samples at 30×*g* for 5 min at 4 °C. This is a **VERY SLOW** spin that is used to remove the remaining hepatocytes and cell clumps that will pellet at the bottom of the tube.
**Step 13**. After centrifugation, collect the supernatant (this is where your immune cells are) with a transfer pipette and transfer it to a new 50 mL conical tube. Try to collect as much supernatant as you can without taking the pellet.
**Step 14**. Repeat steps 12 and 13. (discard the conical tubes with the pellet after each of these two spins-you want the liquid component here).
**Step 15**. Centrifuge the cells solution at 350×*g* for 5 min at 4 °C and remove the supernatant with a vacuum aspirator or transfer pipette (this is waste – the pellet contains the cells you want now).
  Note: If the liver was not perfused well, an Ammonium -Chloride-Potassium (ACK) lysis buffer wash may be done here to eliminate residual red blood cells (RBCs).
  f. Resuspend the pellet in 5 mL of ACK lysing buffer and incubate on ice for 2 min.
  g. Add 30–40 mL of PBS to stop lysis.
  h. Centrifuge at 350×*g* for 5 min at 4 °C and discard the supernatant.
**Step 16**. Resuspend your cells in a flow cytometry buffer until staining for flow cytometry or FACs. The count is required.

### 3.5 Flow cytometry staining

Timing: 2 days.

Once the samples are resuspended in the flow cytometry buffer, the next step is to stain the cells for spectral flow cytometry. Typically activated immune cells continuously produce and release cytokines, which causes low signals. Treatment with Brefeldin A stops intracellular vesicle trafficking and allows for a buildup of the cytokines in the Golgi complex. The accumulation of cytokines increases the detection sensitivity of immune cells during flow cytometry.

**Step 17**. Once a cell count has been performed, dilute cells until cell density is 1 x 10^6^ cells per 100 μL in PBS.
  Note: Our experiments have shown that staining and analyzing at least 5 million cells is ideal when interested in rare populations and it is our recommended method. More cells can be stained if desired. To do so, you can increase the volume in 100μL increments and replace the 500μL listed below with your new desired volume (example: 700 μL for 7 million cells).
**Step 18**. Wash your diluted cell suspension with PBS.
  i. Add 10 mL of PBS.
  j. Centrifuge at 350×*g* for 5 min at 4 °C.
  k. Remove the supernatant.
**Step 19**. Resuspend your cells with 500 μL PBS and vortex.
**Step 20**. Add 1 μL of Zombie UV™ Fixability dye and vortex. Incubate the cells on ice in the dark for 15 min.
**Step 21**. Repeat step 18.
**Step 22**. Resuspend cells in 500 μL of flow cytometry buffer.
**Step 23**. Add 1 μg of Mouse BD Fc Block™ (or equivalent) and vortex. Incubate the cells on ice for 5 min.
**Step 24**. Repeat step 18, replacing PBS with flow cytometry buffer.
**Step 25**. Resuspend each sample in 500 μL of extracellular antibody master mix and vortex.
**Step 26**. Allow cells to stain for 30 min in the dark on ice.
**Step 27**. Add 500 μL of FACS buffer to each sample. Centrifuge the samples at 450×*g* for 5 min at 4 °C and discard the supernatant.
**Step 28**. Wash the cells by resuspending them in 500 μL of FACS buffer again and centrifuge at 450×*g* for 5 min at 4 °C and discard the supernatant.
**Step 29**. Resuspend the samples in 500 μL of BioLegend fixation buffer. Incubate on ice for 20 min in the dark.
  Note: This incubation time may need to be adjusted based on the cytokines being investigated. For our panel, 20 min on ice in the dark was optimal.
**Step 30**. Add 500 μL of BioLegend intracellular staining perm wash buffer and vortex. Centrifuge samples at 1250×*g* for 5 min at 4 °C. Discard the supernatant.
**Step 31**. Repeat step 30 an additional two times.
**Step 32**. Resuspend each sample in 500 μL of intracellular antibody master mix. Incubate on ice in the dark for at least 1 hour.
  Note: This incubation can be left overnight as long as it remains on ice in the dark. Overnight staining has been the preferred method for this protocol to increase sensitivity (Incubation over 10 hours has not been tested).
**Step 33**. Add 500 μL of BioLegend intracellular staining perm wash buffer. Centrifuge samples at 1250×*g* for 5 min at 4 °C. Discard the supernatant.
**Step 34**. Repeat step 33.
**Step 35**. Resuspend samples in 500 μL of FACS buffer. Centrifuge samples at 1250×*g* for 5 min at 4 °C.
**Step 36**. Resuspend samples in 1 mL of FACS buffer. Transfer samples to a flow cytometry tube and keep the samples on ice in the dark until they are ready to run on the spectral flow cytometer.
  Note: Depending on the number of cells being stained and the flow rate of the flow cytometer being used, the final resuspended volume may need to be increased to reduce the concentration. All samples **MUST** be resuspended to the same final volume in order to accurately calculate and compare the total number of cells in each liver, using the formula in the acquisition and analysis section.

## 4 Acquisition and analysis

The samples are now ready to be acquired on the flow cytometer and then analyzed. The panel was designed for use and run on the 5 laser Cytek® Aurora spectral flow cytometer. This instrument has five lasers: ultraviolet (UV, 355 nm), violet (405 nm), blue (488 nm), yellow-green (561 nm), and red (640 nm) lasers. The flow rate during acquisition was set to medium (around 28 μL/min). All samples were set to acquire 5 million events. To calculate the total cell number found in the liver, use the following formula:

$$\frac{Cell\;count}{Volume\;run\;on\;flow\;cytometer}\times Resuspended\;cell\;volume$$

**Fig. 1:**
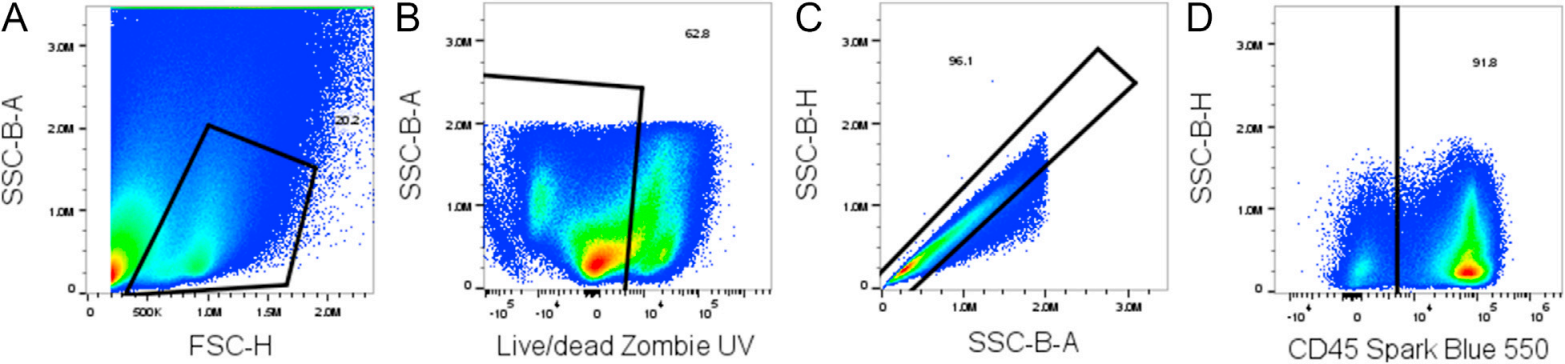
**Flow cytometry initial manual gating strategy**. Gating strategy with showing the initial gating strategy that all following figures underwent. A shows our leukocyte gating, followed by live-dead gating (**B**), single cell gating (**C**), and CD45-positive gating (**D**).

For example, to find the total number of Kupffer cells (KCs) in the liver of a sample, first, use the gating strategy in Fig. 1 to determine their cell count, let's say 28,050 cells, for this example. If using FlowJo, right-click the sample and select inspect. This will tell you the amount of volume run on the flow cytometer for this sample, 140.14 μL for this example. Finally, because we resuspended the samples in 1 mL of FACS buffer before running on the instrument (Step 36), we know our resuspended cell volume is 1000 μL. Therefore, using the formula above, we can determine that the liver contained 200,157 KCs.

This panel has only been tested on the described spectral flow cytometer; however, an equivalent spectral flow cytometer should be sufficient. Data analysis was performed using FlowJo v10.8.1, but FCS Express or any equivalent flow cytometry analysis software could be used. Fig. 1 shows the suggested gating strategy for deep immunophenotyping of liver immune cells.

Note: single color controls must be run on the spectral flow cytometry instrument that you will be using for your flow cytometric acquisition. Data analysis should use Fluorescence Minus One (FMO) controls for proper intracellular cytokine population selection to avoid gating background fluorescence.

## 5 Troubleshooting

### 5.1 Problem 1: low cell numbers

#### 5.1.1 Potential solution

If you encounter low cell numbers and excessive cell death, several factors may contribute to this issue related to the ACK buffer step. It could be due to the ACK buffer step being too long, too concentrated, or too warm. To address this problem, one can use a cooled ACK buffer, shorten the ACK buffer incubation time, keep the ACK buffer incubation on ice, and use less ACK buffer. Additionally, increasing the deoxyribonuclease I concentration may also help to mitigate the cascading death effect.

If there are low cell numbers without excessive cell death, this could result due to a poor release of cells from the extracellular matrix (ECM) during liver digestion. In cases when working with livers with extreme fibrosis, it may be necessary to increase the concentration of collagenase type 4 in the digestion buffer. It may also be advised to increase the amount of digestion buffer the liver is perfused with and increase the water bath incubation time.

### 5.2 Problem 2: No cytokine signal

#### 5.2.1 Potential solution

There may be some optimization required depending on the specific antibodies and fluorophores used for different models. Also, utilizing a different Golgi blocker, such as monensin or bafilomycin A1, may improve signaling depending on the specific cytokines being investigated. For example, Sun *et al*.^5^ had success using monensin for measuring IL-10 production. Finally, not all cytokines, such as the IL-1 family (which lack amino-terminal secretion signal sequences), are processed through the Golgi; therefore, utilization of a Golgi blocker to increase intracellular cytokine concentrations and the signal will not work for these.^6^

## 6 Discussion

**Fig. 2:**
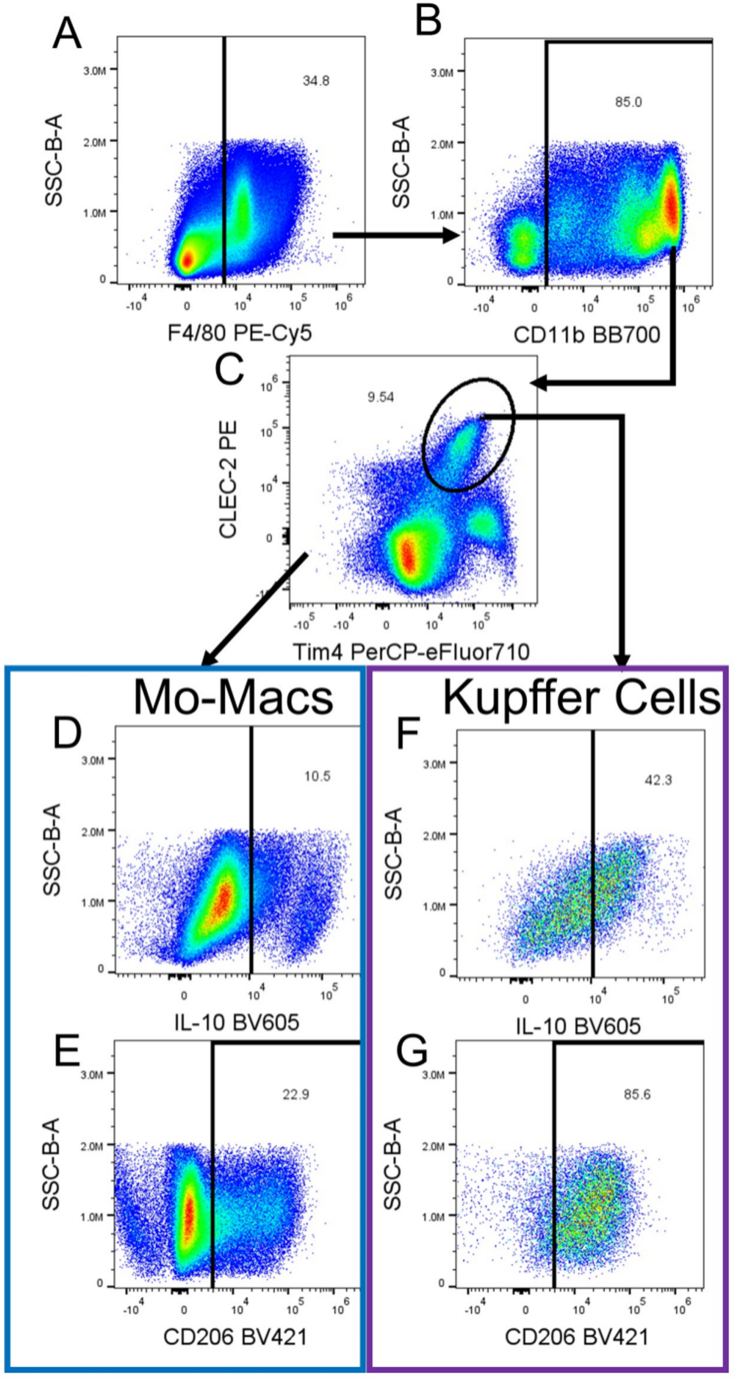
**Flow cytometry macrophage gating strategy.** Gating strategy for identification of monocyte-derived macrophages (Mo-Macs) and Kupffer cells (KCs). Panel A continues from a CD45-positive gate from Fig. 1D and gates on F4/80-positive cells. CD11b-positive cells are selected (B), and KCs and Mo-Macs are differentiated based on Clec2 and Tim4 expression (C). Panels D and F investigate interleukin (IL)-10 expression while panels E and G show CD206 expression for Mo-Macs and KCs, respectively. KCs are defined as CD45^+^F4/80^+^CD11b^+^ and Clec2 and Tim4 dual positive. Mo-Macs are CD45^+^F4/80^+^CD11b^+^ and not Clec2 and Tim4 dual positive. Mo-Macs are split from KCs by utilizing a “Not-Gate”, including all cells that are not gated as Clec2 and Tim4 dual positive in Fig1C.

**Fig. 3:**
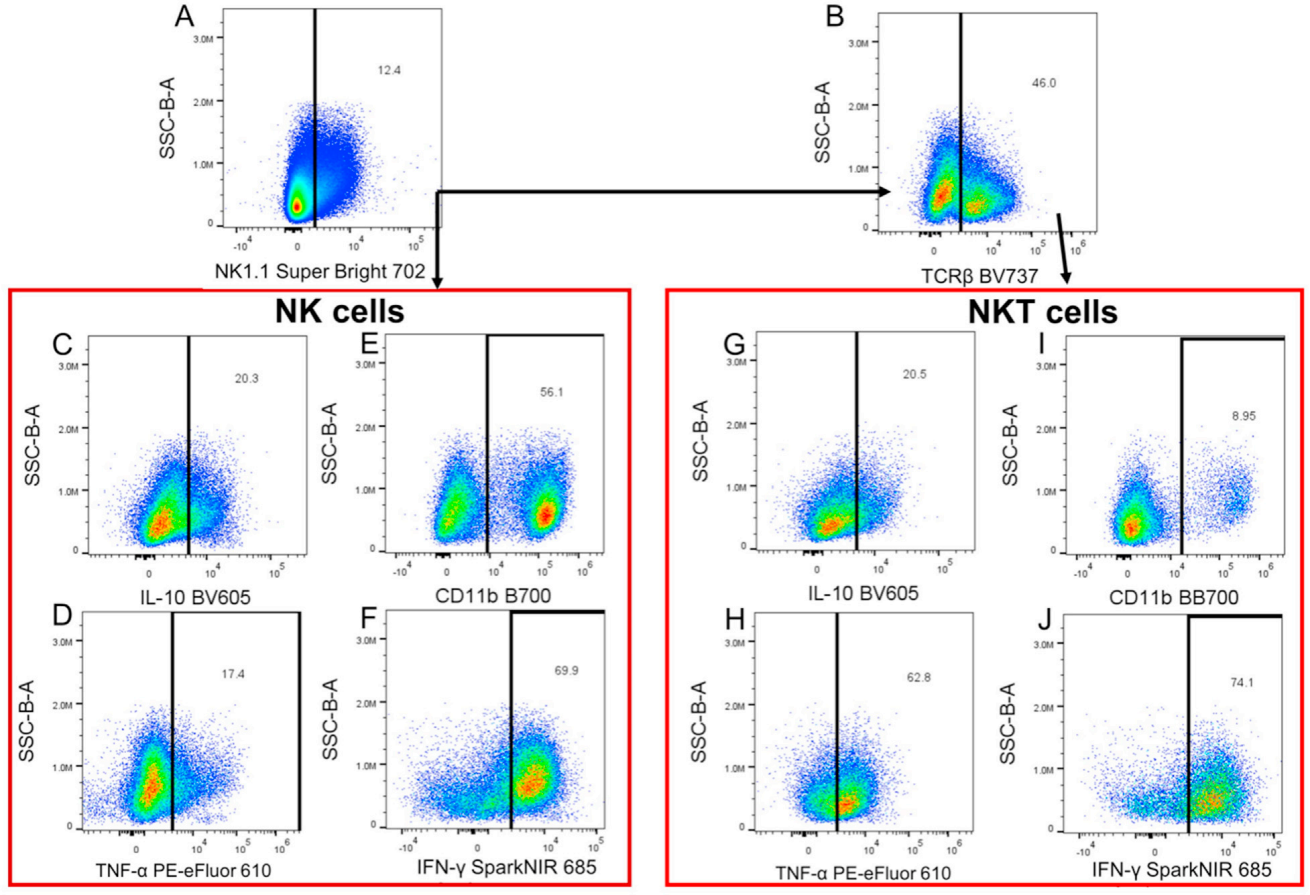
**Flow cytometry NK and NKT cells gating strategy.** Gating strategy for identification of natural killer (NK) and natural killer T (NKT) cells. Panel A continues from a “Not-gate” from Fig.2A, selecting only CD45^+^F4/80^−^ cells. Panel B selects for NKT cells by gating on TCRβ positive NK cells. Panels C and G identify interleukin (IL)-10-positive NK and NKT cells, respectively. Panels E and I identify CD11b-positive NK and NKT cells, respectively. Panels D and H identify TNF-α positive NK and NKT cells, respectively. Panels F and J identify interferon-γ (IFN-γ) expressing NK and NKT cells, respectively. NK cells are identified as CD45^+^F4/80^−^NK1.1^+^ and NKT cells are identified as CD45^+^F4/80^−^NK1.1^+^TCRβ^+^. Fig. 3C–J are used to reveal effector function and maturation/subtyping for NK and NKT cells.

**Fig. 4:**
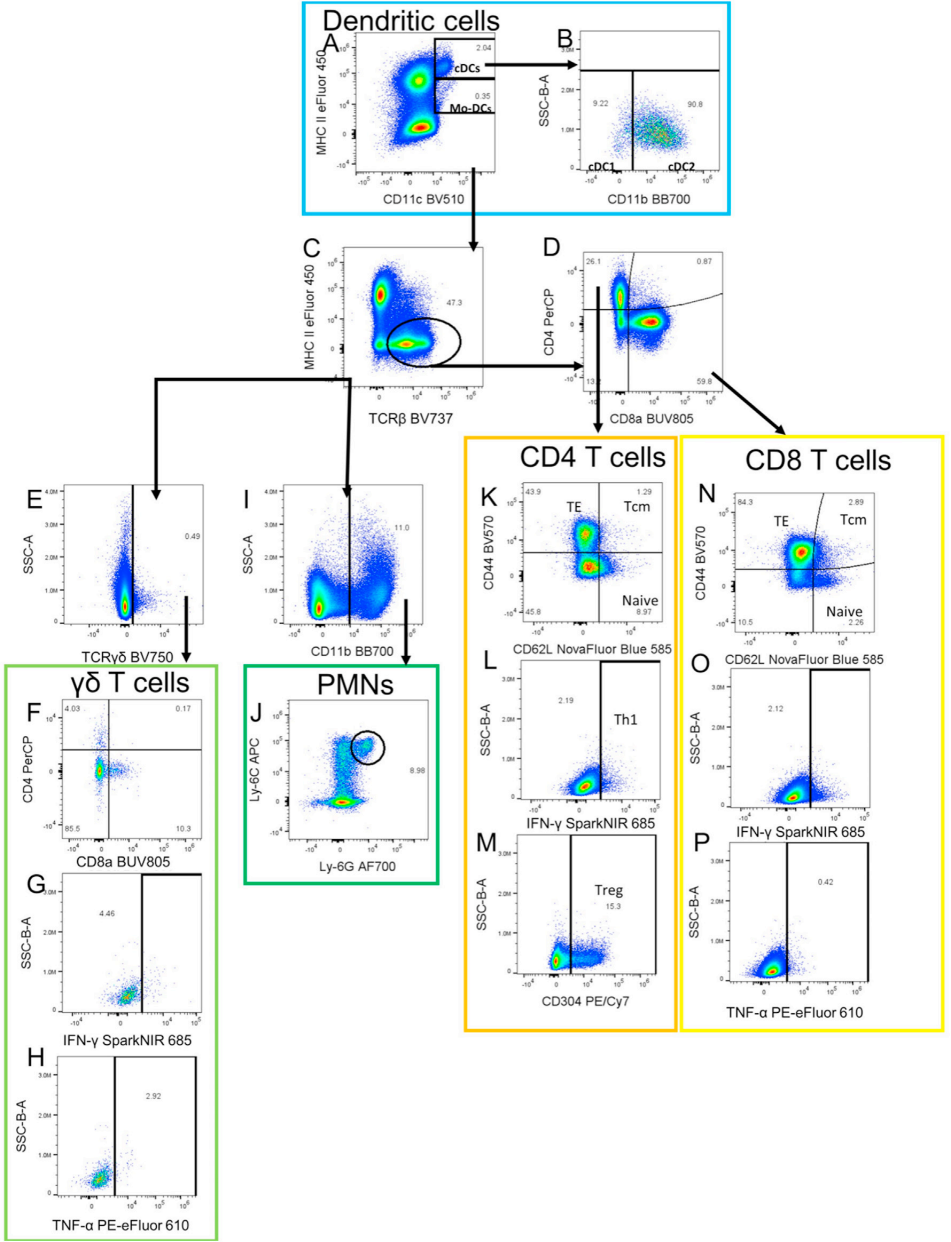
**Flow cytometry dendritic cells, T cells, and PMNs gating strategy.** Gating strategy for identification of type 1 and type 2 conventional dendritic cells (cDC1s (CD45^+^F4/80^−^NK1.1^−^CD11c^+^MHC-II^hi^CD11b^−^) and cDC2s (CD45^+^F4/80^−^NK1.1^−^CD11c^+^MHC-II^hi^CD11b^+^)), monocyte-derived dendritic cells (Mo-DCs) (CD45^+^F4/80^−^NK1.1^−^CD11c^+^MHC-II^int^), CD4 T cells (CD45^+^F4/80^−^NK1.1^−^TCRβ^+^CD4^+^), CD8 T cells (CD45^+^F4/80^−^NK1.1^−^TCRβ^+^CD8^+^), effector T cells (TE) (CD45^+^F4/80^−^NK1.1^−^TCRβ^+^CD44^+^CD62L^−^), central memory T cells (Tcm) (CD45^+^F4/80^−^NK1.1^−^TCRβ^+^CD44^+^CD62L^+^), naive T cells (CD45^+^F4/80^−^NK1.1^−^TCRβ^+^CD44^−^CD62L^+^), T helper type 1 (Th1) (CD45^+^F4/80^−^NK1.1^−^TCRβ^+^CD4^+^IFN-γ^+^), Treg (CD45^+^F4/80^−^NK1.1^−^TCRβ^+^CD4^+^CD304^+^), γδ T cells (CD45^+^F4/80^−^NK1.1^−^TCRγδ^+^), and polymorphonuclear cells (PMNs) (CD45^+^F4/80^−^NK1.1^−^CD11b^+^Ly-6G^+^Ly-6C^+^). Panel A continues from a “Not-gate” from Fig.3A, selecting only CD45^+^F4/80^−^NK1.1^−^ cells and identifying cDCs and Mo-DCs based on CD11c and MHC-II expression. CD11b expression is used to separate cDC1s from cDC2s (B). C results from a “Not-Gate” excluding both cDC and Mo-DC gates and identifies TCRβ^+^ T cells based on TCRβ^+^MHC-II^−^ expression (C). CD4 and CD8 expression to identify CD4 and CD8 T cells (D). E arises from a “Not-Gate” from Fig.4C to ensure no TCRβ^+^ T cells are in the γδ T cell population and selects for γδ T cells (E). CD4 and CD8 expression is used to subtype γδ T cells (F). γδ T cell IFN-γ expression is investigated (G). TNF-α expression of γδ T cells is also investigated (H). A “Not-Gate” from Fig. 4C is used, and gates on CD11b-positive cells (I). Ly-6G and Ly-6C expressing cells are gated on to identify PMNs (J). CD44 and CD62L expression is used to identify TEs, Tcms, and Naive CD4 T cells (K). IFN-γ expressing CD4s are identified as Th1s (L). CD304 expressing CD4 T cells are identified as Tregs (M). CD44 and CD62L expression is used to identify TEs, Tcms, and Naive CD8 T cells (N). IFN-γ (O) and TNF-α (P) are investigated to determine CD8 effector function.

The methodology outlined above can identify several hepatic immune populations and elucidate the cytokines being secreted by each. The gating strategies shown in Fig. 1, Fig. 2, Fig. 3, Fig. 4 identify polymorphonuclear leukocytes, monocyte-derived macrophages, KCs, NK cells, NKT cells, type 1 conventional dendritic cells (cDC1s) and cDC2s, monocyte-derived dendritic cells, effector T cells (TE), central memory T cells (Tcm), γδ T cells, and naive CD4 and CD8 αβ T cells. Further subtyping of KCs and macrophages via CD206 allows us to identify M2-like/pro-resolving macrophages, and we are further able to determine how many cells are actively expressing IL-10.^7^ NK and NKT cells can be further analyzed by maturation (based on CD11b expression) and their expression of IL-10, TNF-α, and IFN-γ.^8^ CD4 and CD8 T cells are identified by CD44 and CD62L expression as TE, Tcm, or naive T cells. IFN-γ staining defines CD4 T cells as Th1 cells, while CD304 is used to measure how many regulatory T cells are present.^9^ IFN-γ and TNF-α staining elucidate CD8 T cell activity.

Immunophenotyping of the liver has been described previously in other methods. However, these methods either lack cytokine staining or have limitations in immune cell identification, which ultimately hinders the accurate characterization of the hepatic immune compartment and determination of immune cell effector function.^1^ The methods outlined here provide a tool for deep immunophenotyping that can be used for various liver diseases. This in-depth immune characterization can help elucidate pathological immune changes associated with several liver diseases. While surface staining can help identify different cell types in these disease states, cytokine staining is required to determine their effector functions and interactions with the surrounding cells. Cytokine staining for flow cytometry is inherently tricky as cytokines are continuously exported from active immune cells, leading to a weak signal when immune cells are analyzed during flow cytometry. Historically, this has been solved by isolating immune cells *ex vivo* and treating them with a Golgi blocker to stop the exportation of these cytokines, increasing the signal strength observed during data analysis. However, the *ex vivo* treatment of immune cells removes them from the *in vivo* signaling they would normally receive in the animal and may lead to an inaccurate representation of their functioning during disease. Therefore, the method outlined here permits a more accurate characterization of the hepatic immune environment compared to the traditionally used *ex vivo* Brefeldin A administration. By preserving the immune cells in their *in vivo* state, this protocol provides a more faithful representation of their functioning within the context of disease.

## Ethical approval

All animal studies were conducted following VCU institutional policies under the ethical approval number AD10000319.

## Authors’ contributions

Grayson W. Way: Conceptualization, Methodology, Investigation, Formal analysis, Validation, Writing - Original Draft, Reviewing and Editing. Hongkun Lu: Visualization, Writing - Reviewing and Editing. Xuan Wang: Investigation. Derrick Zhao: Investigation. Carmen Camarena: Writing - Original Draft. Devanand Sarkar: Resources. Rebecca K. Martin: Conceptualization, Methodology, Validation, Formal analysis, Resources, Writing- Original Draft, Supervision. Huiping Zhou: Resources, Supervision, Project administration, Funding acquisition. All authors approved the final version for publication.

## Declaration of competing interest

Huiping Zhou is an associate editor for *Liver Research* and was not involved in the editorial review or the decision to publish this article. All authors declare that there are no competing interests.
